# Enhancing protein inter-residue real distance prediction by scrutinising deep learning models

**DOI:** 10.1038/s41598-021-04441-y

**Published:** 2022-01-17

**Authors:** Julia Rahman, M. A. Hakim Newton, Md Khaled Ben Islam, Abdul Sattar

**Affiliations:** 1 School of Information and Communication Technology, Griffith University, Southport, Australia; 2 Institute of Integrated and Intelligent Systems, Griffith University, Southport, Australia

**Keywords:** Protein structure predictions, Computational models

## Abstract

Protein structure prediction (PSP) has achieved significant progress lately via prediction of inter-residue distances using deep learning models and exploitation of the predictions during conformational search. In this context, prediction of large inter-residue distances and also prediction of distances between residues separated largely in the protein sequence remain challenging. To deal with these challenges, state-of-the-art inter-residue distance prediction algorithms have used large sets of coevolutionary and non-coevolutionary features. In this paper, we argue that the more the types of features used, the more the kinds of noises introduced and then the deep learning model has to overcome the noises to improve the accuracy of the predictions. Also, multiple features capturing similar underlying characteristics might not necessarily have significantly better cumulative effect. So we scrutinise the feature space to reduce the types of features to be used, but at the same time, we strive to improve the prediction accuracy. Consequently, for inter-residue real distance prediction, in this paper, we propose a deep learning model named scrutinised distance predictor (SDP), which uses only 2 coevolutionary and 3 non-coevolutionary features. On several sets of benchmark proteins, our proposed SDP method improves mean Local Distance Different Test (LDDT) scores at least by 10% over existing state-of-the-art methods. The SDP program along with its data is available from the website https://gitlab.com/mahnewton/sdp.

## Introduction

Protein structure prediction (PSP) is recognised as one of the long standing unsolved problem in bio-informatics, biophysics, and structural biology^1^. A protein’s function depends on its three dimensional *native structure* that has the minimum kinetic energy. PSP is thus a crucial step in developing life-saving medicines, in designing novel enzymes, and in therapeutic science. Prediction of the native structure of a protein directly from its amino acid sequence is a complex procedure since the conformational search space is astronomical and the energy function is by and large unknown^2^.

Energy functions such as CHARMM^3^ and AMBER^4^ are based on molecular dynamics and have computed energy components from chemical bonds, bond angles, dihedral angles, van der waals forces, and electrostatic forces. However, these energy functions have so far led to poor prediction of protein structures. Moreover, neither they are good in capturing long range inter-residue or inter-atomic interactions nor are computationally efficient. Knowledge based energy functions have statistically derived structural features from available experimentally verified proteins. Such energy functions are computationally cheaper since they are mostly at the residue level. Consequently, residue–residue contact (whether distance is less than $8\,\AA$) prediction algorithms have been developed and predicted contacts have been used as geometric constraints in ab initio PSP search^2,5,6^. Contact maps have also been used in transforming into inter-residue distances by methods such as CONFOLD^7^, CONFOLD2^8^, and DESTINI^9^. However, contact maps suffer from their inability to distinguish distances that are beyond $8\,\AA$ and also from the fact that on an average more than $92\%$ residue pairs are not in contact^10^. In this context, inter-residue distance maps are more informative than residue–residue contact maps since distances are real numbers while contacts are boolean values. Recently, AlphaFold^11^ and trRosetta^12^ have shown promising results using inter-residue distances during search. Inter-residue distances have also been used in threading approaches^13^. Note that both in contact and distance maps, residues are represented by their $C_\beta$ atoms ($C_\alpha$ for Glycine) since side chains are critical for more accurate protein structure construction^14^.

Early distance map prediction methods use shallow neural networks^15–18^ or from homologous proteins^19^. In distance maps, distances could be represented by binned ranges or by real values. Recently, binned ranges or distograms have been predicted by AlphaFold^11^ and other methods^12,20^, mainly using classification based deep learning algorithms. Real valued distance prediction^16,21^ has been addressed as a regression problem by Generative Adversarial Network-based method (GANProDist)^22^. Recent distance map prediction methods PDNET^23^ and LiXu^24^ (we name it after the author names since it has no original name) predict both real-valued and binned distances while another recent method DeepDist^25^ predicts real-valued distances. Because of the vital role of distance maps in template-free or Free Modelling (FM) structure prediction, the Critical Assessment of protein Structure Prediction (CASP) organisers have introduced a new challenge category “inter-residue distance prediction” in CASP-14^26^. PSP has obtained significant progress lately via distance map based energy functions. However, further progress needs more accurate inter-residue distance prediction since the quality of a predicted protein structure highly depends on the accuracy of the distance prediction.

State-of-the-art distance or contact map prediction algorithms^11,12,20,23,25,27–29^ are largely based on Convolutional Neural Networks (CNN)^30^ or Residual Networks (ResNet)^24,31^. Moreover, these methods predominantly use multiple sequence alignment (MSA) based coevolutionary features. MSA based features have been used for long in contact map prediction^28,32–35^ and since CASP-11, also in distance map prediction^22,25^. However, most popular MSA based features such as Covariance-Matrix^25^, Precision Matrix^25,29^, Pseudolikelihood Maximization Matrix^25^, Compressed Covariance-Matrix^28^, Reduced Precision Matrix^28,29^ take huge amounts of memory. Also, MSA based features have weaknesses particularly with proteins that have not many homologous sequences. Non-coevolutionary sequence based features e.g. Position-Specific Scoring Matrix (PSSM)^36^ and Solvent Accessibility (ACC)^37^ have been used to deal with such proteins^25^. Nevertheless, despite the progress made in distance prediction algorithms, prediction of large distances and distances between residues that have long sequence separation length still remains challenging. To overcome this, very recent distance prediction algorithms have used more and more coevolutionary and non-coevolutionary features and more complex neural networks. For example, PDNET^23^, DeepDist^25^, and LiXu^24^ use respectively 3, 5, and 3 types of coevolutionary and 4, 7, and 3 types of non-coevolutionary features. Also, DeepDist^25^ and LiXu^24^ use ensembles of 4 and 6 ResNets respectively.

In this paper, we argue that the more the types of features, the more the kinds of noises introduced and then the deep learning model has to overcome the noises to improve the accuracy of the predictions. Also, multiple features capturing similar underlying characteristics might not necessarily have significantly better cumulative effect. So we scrutinise the feature space to reduce the types of features being used but at the same time, we strive to improve the prediction accuracy. Our approach is inspired by Occam’s rajor principle and by the improved performance obtained by simpler models in backbone angle prediction^38^. In this paper, for inter-residue real distance prediction, we propose a dilated ResNet based deep learning model, which uses fewer types of MSA and sequence based features than existing such methods. In particular, our model uses 2 coevolutionary types of features CCMPred^33^ and FreeContact^39^, and 3 non-coevolutionary types of features PSSM, ShannonEntropy^34^, and Seven Physicochemical Properties (7PCP)^40^. The 7PCP features include steric parameter (graph shape index), hydrophobicity, volume, polarisability, isoelectric point, helix probability, and sheet probability. On several sets of benchmark proteins, our proposed algorithm improves mean Local Distance Different Test (LDDT) scores at least by 10% over existing state-of-the-art methods. Our proposed algorithm is named Scrutinised Distance Predictor (SDP). The SDP program along with its data is available from the website https://gitlab.com/mahnewton/sdp.

## Methods

We describe the benchmark datasets, input features, and ResNet architecture and implementation of our proposed SDP method.

### Benchmark datasets

We have initially taken the same dataset used by MapPred^28^ as well as SPOT-1D^41^. This dataset contains 12,450 proteins. These proteins were culled from PISCES^42^ on February 2017 and curated by satisfying the constraints of high resolution $$ < 2.5\,\AA $$, R-free $$ < 1$$, and pairwise sequence identity less than 25% similarity according to BlastClust^43^. However, we have performed some additional cleaning on the dataset. For example, similar to someother work^9,35,41,44^, we have ignored the proteins which have less than 25 or more than 700 residues in their sequences. During additional cleaning, we have found 7145 proteins which have the exact amino acid sequences in both Fasta and PDB files. The rest 1910 proteins are selected by taking amino acid sequences from PDB where Fasta sequence has some additional residues at the beginning or at the end of the sequence. The finally filtered dataset in total contains 9055 proteins. From these proteins, a random set of 680 proteins is selected as the validation set and the remaining 8375 proteins are considered as the training set for our proposed model.

To evaluate the effectiveness of our proposed model, we have used three blind test sets: 31 free modelling (FM) targets from CASP13^45^ released in 2018, 131 CAMEO-HARD targets^46^ released from 8th December 2018 to 1st June 2019, and another 144 CAMEO-HARD targets^46^ released from 8th August 2020 to 6th February 2021. These three datasets are denoted by CASP13.31, CAMEO.131, and CAMEO.144 respectively. In case of CAMEO.144, those 144 proteins are obtained from a set of 409 candidate proteins after applying cleaning and excluding the sequences having more than 25% sequence similarly with the training data. For this similarity removal, we have used CD-HIT^47^ and BLAST+^48^ with e-value 0.001. The other two datasets are used as the test datasets by trRosetta^12^ and PDNET^23^.

### Input features

In SDP, we have aggregated five informative features: (1) CCMPred^33^, (2) FreeContact^39^, (3) PSSM^36^, (4) ShannonEntropy^34^ and (5) 7PCP^40^. All of these are easy to generate and take less memory. CCMPred and FreeContact are co-evolutionary features which capture covariance strength of all residue-residue positions in MSA. Sequential features such as PSSM calculates the occurrence of each residues in the MSA sequences and Shannon Entropy extracts the information about the variability in each residue position. Thus, these four features all are generated from MSA. So we try to find other features that do not realy on MSA and rather capture more information about protein structures. We do not consider HHM^49^ or HMM profiles^50^, and Contact Potential^34^ because they are also extracted from MSA. We do not use coevolutionary features such as Precision Matrix^25,29^, Pseudolikelihood Maximization Matrix^25^, Compressed Covariance-Matrix^28^, and Reduced Precision Matrix^28,29^ because these are expensive in terms of memory and time. We choose 7PCP rather than ACC because ACC represents only one property related to hydrophobicity whereas 7PCP contains 7 physicochemical properties. We also consider 8-class secondary structures (SS) predicted by SSpro8^51^ and show experimental results but the results are not satisfactory. To generate MSA, we use hh-suite3^52^ from Uniclust30 database of June 2020^53^. Among our selected 5 features, for PSSM, Shannon Entropy and 7PCP, we need to transform 1D features into 2D-features by tiling and transposed tiling. SDP in total has 62 2D channels.

### ResNet architecture

**Figure 1:**
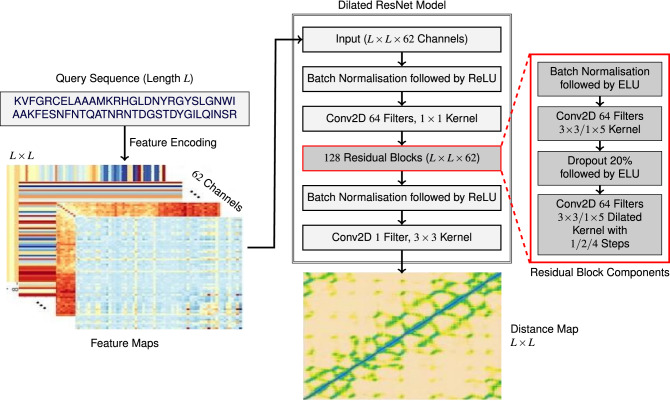
Our proposed dilated ResNet model.

Inspired by the use of ResNet and Dilated ResNet models by RaptorX^20^, AlphaFold^11^, trRosetta^12^, and PDNET^23^ for binned or real-valued distance prediction, we use two dimenionsional Dilated ResNet shown in Fig. 1 for our proposed SDP method. The ResNet in SDP takes generated 2D-features and feeds them to a batch normalisation layer followed by a rectified linear unit (ReLU) activation function. Then, SDP has a 2D convolution layer with $1\times 1$ kernel, a layer of 128 residual blocks, another batch normalisation layer followed by a ReLU function, and finally another 2D convolutional layer with $3\times 3$ kernel. The last 2D convolutional layer produces the inter-residue distance map. In the layer having 128 residue blocks, each residual block contains a batch normalisation layer, an exponential linear unit (ELU) activation layer, a 2D convolution layer, a dropout layer with drop out rate 20%, and another 2D convolutional layer. The 2D convolution layers have alternating between $3\times 3$ and $1\times 5$ kernels with dilation. The dilation cycle in the second 2D convolutional layers alternate by 1, 2, and 4 steps. The last 2D convolutional layer producing the distance map has 1 filter while all other 2D convolutional layers in our model have 64 filters and “he normal” kernel initialiser. As is done in AlphaFold^11^ and PDNET^23^, we add zero padding of width 5 to all slides of input features and generate cropped samples of $128\times 128$ randomly from the input. However, after prediction, we do not do any such types of padding or cropping in the predicted values.

As noted before, inter-residue real distance prediction is considered as a regression problem. For a regression problem, it is challenging to pick an appropriate loss function, which can led to prediction of real values as correctly as possible. Commonly used loss functions such as MAE or Mean Square Error (MSE) have the tendency to focus on the long distances because they create higher loss values. However, in the real-valued inter-residue distance prediction problem, shorter distances are more meaningful than longer ones in terms of the usefulness in constructing protein structures. To address this problem, GANProDist^22^ transforms real-valued distances in the $[-1,1]$ interval and achieves large gradients for actual distance in 4–16 $\AA$. On the other hand, DeepDist^25^ predicts inter-residue real-valued distances only less than 16 $\AA$ by using an ensemble of four ResNets with MSE loss function. Moreover, PDNET^23^ uses the reciprocal log cosh loss function to convert longer distances into shorter ones and vice versa. In this paper, we have chosen the log cosh loss function because of it’s capability to deal with both short and long distances. However, we also transform our actual distance values into reciprocal distances by using $f(d) = 100/d^2$ function before applying the deep learning model on it. Then, after prediction, we apply the inverse function of *f*(*d*). Eventually this is somewhat similar to the effect of the reciprocal log cosh function.

### ResNet implementation

We have implemented our proposed model in Python(version 3.7.6) language using the Keras library. The data generator module of Keras is used in loading the features batch by batch. Our model is trained with batch size 2 and the number of epochs for training 100. RMSprop optimiser is used with the default learning rate of 0.001. We run our programs on NVIDIA Tesla V100-PCIE-32GB machines. One epoch of the training takes around 30 min.

## Results

To show the impact of various components of the proposed SDP method, we create a number of SDP variants and compare them. We then compare SDP with the current state-of-the-art distance map predictor methods. For comparison, we mainly use MAE values and lDDT^54^ scores computed from distance predictions.

Table 1 shows percentages of residue pairs having distances within ranges [*l*, *h*) where $h-l = 4$. We show prediction results for inter-residue distances up to $36\,\AA$ and thus cover more than $59\%$ residue pairs while existing methods such as RaptorX^20^ and DeepDist^25^ consider distances up to $16\,\AA$ and cover less than $18\%$ residue pairs. In this context, we define distances below $16\,\AA$ as *short distances* and distances below $36\,\AA$ as *long distances*; short distances are naturally a subset of long distances. Note that while training the ResNet, depending on our target to achieve short or long distance prediction, we might use all possible residue-pairs or those having certain maximum distances. Later, in appropriate sections, we will mention exactly which residue-pairs are used in training of which model. We are interested in improving long distance prediction.

**Table 1:** Percentages of residue pairs having distances within [*l*, *h*) ranges.

Test Dataset	00-04	04-08	08-12	12-16	16-20	20-24	24-28	28-32	32-36	0-16	0-36
CASP13.31	0.04	3.25	5.72	8.47	10.06	10.5	10.02	8.92	7.56	17.48	64.54
CAMEO.131	0.03	2.38	4.37	6.77	8.54	9.55	9.84	9.49	8.69	13.55	59.66
CAMEO.144	0.04	3.15	5.77	8.85	10.98	11.97	11.88	10.88	9.29	17.81	72.81

### Determining best settings

In SDP variants, we consider 6 features CCMPred^33^, FreeContact^39^, PSSM^43^, ShannonEntropy^34^, 7PCP^40^, and 8-state SS^51^. These features have respectively, 1, 1, 44, 2, 14, and 16 channels. Among these features, we consider CCMPred, FreeContact, PSSM as the three core features. Then, we add ShannonEntropy to see its effectiveness empirically. Lastly, we consider adding one or both of 7PCP and SS features to see their separate or combined effect. For the ResNet layer having residual blocks, we consider either 64 or 128 blocks. Most existing methods use 128 residual blocks, but we empirically evaluate using fewer blocks. In total, we have 10 SDP variants, which are listed below.

**CF64, CF128:** Core Features (CCMPred, FreeContact, and PSSM) and 64 or 128 residual blocks
**SE64, SE128:** ShannonEntropy with Core Features and 64 or 128 residual blocks
**PC64, PC128:** 7PCP with ShannonEntropy plus Core Features and 64 or 128 residual blocks
**SS64, SS128:** SS with ShannonEntropy plus Core Features and 64 or 128 residual blocks
**SSPC64, SSPC128:** SS and 7PCP with ShannonEntropy plus Core Features and 64 or 128 residual blocks

Note that considering short and long distance predictions, various subsets of residues could be used in training these 10 variants. However, to select one best model without cluttering the comparison landscape, we just show the results where all residue-pairs have been used in training the 10 variants. Further, note that we show results only for the CAMEO.144 datasets but the results are similar for the validation datasets and the other test datasets.

**Figure 2:**
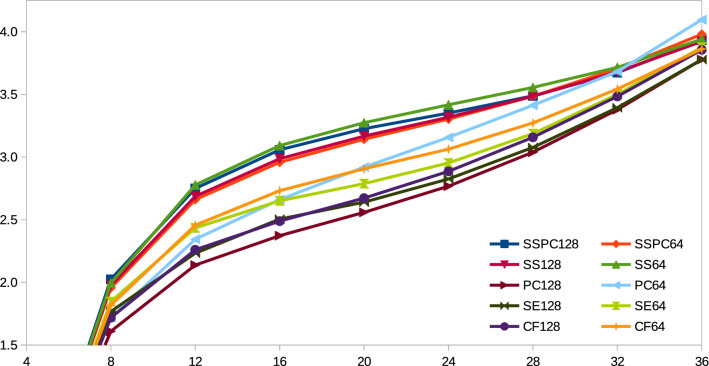
MAE values (y-axis) obtained by SDP variants over inter-residue distances in [0, *h*) where *h* is a threshold (x-axis).

Figure 2 shows the MAE values obtained by the SDP variants over inter-residue distances in the ranges [0, *h*) where *h* is a threshold in multiples of $4\,\AA$. As we can see, in general, the MAE values increase for all variants as more distant residue pairs are included. Also, 128 residual blocks are better than 64 blocks except in SSPC variants. Adding ShannonEntropy with the three core features improves the MAE values. Then, PC128 performs better than SE128 while PC64 is better than SE64 only up to residue pair distances of $16\,\AA$. So addition of the 7PCP features in general improves the MAE values with 128 residual blocks. However, addition of SS features in general causes degradation of the MAE values. Overall, PC128 appears to be the best performer among the 10 SDP variants. So, henceforth, we will use PC128 variant that uses 7PCP, ShannonEntropy, CCMPred, FreeContact, and PSSM features as our main SDP algorithm.

For the selected SDP algorithm, as discussed above, we have the following five variants depending on our target of short or long distance prediction. These five variants use the same 5 features and the same ResNet architecture, but only the training datasets are different for them. We will later compare the best ones from the five variants with the state-of-the-art inter-residue distance prediction methods.

**SDP-L:** Targeting long distance prediction, uses our training and validation proteins as described exactly before.
**SDP-X:** Targeting long distance prediction, uses the training and validation proteins of PDNET^23^, instead of our training and validation proteins. This allows us to see the effectiveness of our features and the ResNet model over various datasets.
**SDP-Y:** Targeting short distance prediction, uses value $16\,\AA$ as the distance between each two residues that are actually more than $16\,\AA$ apart. $16\,\AA$ is a distance threshold used in RaptorX^20^ and DeepDist^25^.
**SDP-S:** Targeting short distance prediction, customises the loss function to ignore residue pairs that are actually more than $16\,\AA$ apart. Compared to the approach in SDP-Y, this is another way to target short distance prediction.
**SDP-Z:** Targeting short distance prediction, uses the training and validation proteins of PDNET^23^, instead of our training and validation proteins. Like SDP-S, this customises the loss function to ignore residue pairs that are actually more than $16\,\AA$ apart. Like SDP-X, this allows us to see the effectiveness of our features and the ResNet model over various datasets.

Note that for training and validation, MSA used by PDNET, SDP-X, and SDP-Z is based on Uniclust30 database of August 2018^55^. For training and validation of SDP-S, SDP-L, and SDP-Y, MSA is based on Uniclust30 database of June 2020^53^. For all testing proteins from CASP13.31, CAMEO.131, and CAMEO.144 regardless of the SDP variants, MSA is based on Uniclust30 database of June 2020^53.^

### Comparison with state-of-the-art distance predictors

As noted before SDP uses 2 coevolutionary and 3 non-coevolutionary features such as CCMPred, FreeContact, PSSM, ShannonEntropy, and 7PCP. We compare SDP with most recent inter-residue distance prediction methods PDNET^23^, DeepDist^25^, and LiXu^24^. We briefly describe them below. We could not compare SDP with GANProDist^22^ because its model or program is not available and its online server cannot generate distance maps for the proteins with more than 500 or fewer than 40 residues.

**DeepDist:** It works mostly in short distance ($\le 16\,\AA$) prediction. It uses 5 coevolutionary and 7 non-coevolutionary features such as Covariance-Matrix, Precision Matrix, Pseudolikelihood Maximisation Matrix, CCMPred, Contact Potential, PCC, PSSM, ShannonEntropy, ACC, Mutual Information^25^, Normalised Mutual Information^25^, and Joint Entropy^25^. Note that DeepDist generates MSA from 6 sources such as Uniclust30 of October 2017^56^, Uniref90 of April 2018^57^, Metaclust50 of January 2018^58^, and also a customised database that combines Uniref100 of April 2018^59^, metagenomics sequence databases of April 2018, and NR90 database of 2016. DeepDist uses an ensemble of 4 ResNets.
**PDNET:** It works well with large distances ($\ge 16\,\AA$). It uses 3 coevolutionary and 4 non-coevolutionary features such as CCMPred, Contact Potential, FreeContact, PSSM, SS (3class), ACC, and ShannonEntropy. As noted before, it generates MSA from Uniclust30 database of August 2018^55^. PDNET uses just one ResNet.
**LiXu:** It works mostly in short distances ($\le 15 \,\AA$) prediction. It uses 3 coevolutionary and 3 non-coevolutionary features such as amino acid sequence represented by one-hot encoding, sequence profiles generated by MSA, secondary structure and solvent accessibility predicted from the sequence profiles^37^, co-evolution information including mutual information^25^, and CCMpred output matrices. For MSA and sequence profile generation, it uses uniclust30 (dated in August 2018), uniclust30 (dated in October 2017), uniref90 (dated in March 2018), and metaclust (dated in June 2018) as sequence libraries. Moreover, it uses an ensemble of 6 ResNets with some kind of squared errors as loss functions.

As noted before, for all testing proteins from CASP13.31, CAMEO.131, and CAMEO.144, we generate MSA using Uniclust30 database of June 2020^53^. We use the same MSA for the testing proteins when we run DeepDist and PDNET.

We present our results in two ways: first, with PDNET^23^ and DeepDist^25^ in details and then, with LiXu^24^ briefly. The LiXu program is not available and we compared its published results with our results using the same distance metrics that LiXu uses.

Let $D_{ij}$ be the actual distance between residues with indexes *i* and *j* and $S_{ij}$ the sequence separation length $|i-j|$.

#### Comparison with PDNET and DeepDist

**Figure 3:**
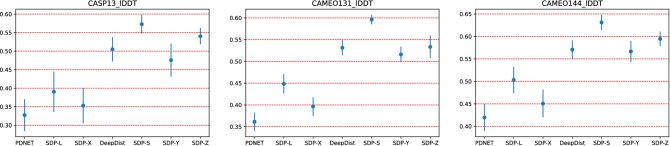
95% confidence interval plots for lDDT scores of PDNET, DeepDist and SDP methods.

Table 2 shows the mean lDDT values for PDNET, DeepDist and SDP methods over all residue pairs in each dataset. As per DISTEVAL^26^, lDDT scores are the most effective metrics to evaluate predicted real-valued distances. As we see from the table, SDP-L among PDNET, SDP-X, and SDP-L obtains the best mean lDDT score while SDP-S among DeepDist, SDP-Y, SDP-Z, and SDP-S obtains the best mean lDDT score. Among all 7 competing methods, SDL-S obtains the best mean lDDT score. Figure 3 shows the 95% confidence interval plots for the lDDT scores of PDNET, DeepDist, and SDP methods. Any overlapping of the confidence interval means the differences are not statistically signficant. As we see from the charts, SDP-L is significantly better than PDNET in CAMEO.131 and CAME.144 proteins but not in CASP13.31 proteins. Moreover, SDP-S is significantly better than DeepDist in all three datasets. DeepDist is also significantly better than PDNET in all three datasets.

**Table 2:** Comparison of PDNET, DeepDist, and SDP methods in terms of mean lDDT values over all residue pairs in each dataset.

Test	Prediction	Mean	MAE for $D_{ij}<16$			MAE for $D_{ij}<36$		
Dataset	Method	lDDT	$S_{ij}\ge 1$	$S_{ij}\ge 12$	$S_{ij}\ge 24$	$S_{ij}\ge 1$	$S_{ij}\ge 12$	$S_{ij}\ge 24$
CASP13.31	PDNET	0.326	3.89	5.37	5.77	4.55	5.12	5.41
	SDP-X	0.352	3.33	4.55	4.86	$*$**3.37**	4.46	4.67
	SDP-L	**0.393**	**3.02**	**4.09**	**4.34**	3.88	$*$**4.37**	$*$**4.58**
	DeepDist	0.503	1.73	1.94	1.97	6.39	**7.12**	**7.33**
	SDP-Y	0.475	1.74	2.17	2.20	**6.36**	7.16	7.38
	SDP-Z	0.540	1.66	1.92	2.01	8.40	9.48	9.58
	SDP-S	$*$0.569	$*$**1.49**	$*$**1.82**	$*$**1.85**	7.88	8.91	9.25
CAMEO.131	PDNET	0.361	4.00	5.94	6.59	4.75	5.52	5.93
	SDP-X	0.396	3.14	4.52	5.04	3.79	4.29	4.55
	SDP-L	**0.451**	**2.67**	**3.8**	**4.11**	$*$**3.64**	$*$**4.23**	$*$**4.37**
	DeepDist	0.527	1.53	1.88	1.92	6.92	7.93	8.21
	SDP-Y	0.516	1.53	1.93	1.97	**6.68**	**7.75**	**8.02**
	SDP-Z	0.559	1.56	1.92	1.93	9.32	10.90	10.62
	SDP-S	$*$**0.596**	$*$**1.36**	$*$**1.67**	$*$**1.73**	8.26	9.6	9.96
CAMEO.144	PDNET	0.420	3.27	4.71	5.15	3.99	4.56	4.96
	SDP-X	0.450	2.78	3.92	4.26	3.47	3.98	4.25
	SDP-L	**0.506**	**2.25**	**3.06**	**3.23**	$*$**3.31**	$*$**3.81**	$*$**4.15**
	DeepDist	0.570	1.48	1.63	1.66	6.41	7.50	8.32
	SDP-Y	0.567	1.37	1.71	1.75	**6.33**	**7.49**	**8.21**
	SDP-Z	0.595	1.49	1.71	1.68	9.10	10.04	10.85
	SDP-S	$*$**0.631**	$*$**1.24**	$*$**1.54**	$*$**1.55**	7.79	9.24	10.1

Also, comparison in terms of MAE values for short and long distances and various sequence separation lengths. The column-wise bold values denote the best lDDT or MAE values over the competing methods {PDNET, SDP-X, SDP-L} or {DeepDist, SDP-Y, SDP-Z, SDP-S} for the same dataset. The column-wise starred values denote the best lDDT or MAE values over all the 6 competing methods for the same dataset. For lDDT, the larger the better while for MAE, the smaller the better.

In terms of MAE values, the performance difference between SDP-L and PDNET is statistically significant as per t test with 95% significance level (p values are 0.0 for all datasets) and so is also the difference between SDP-S and DeepDist. Table 2 also shows the MAE values for PDNET, DeepDist, and SDP methods for residue pairs that are short and long distance apart and have various sequence separation length. Although Table 2 shows results for all combinations, we mainly compare SDP-Y, SDP-Z, and SDP-S with DeepDist since DeepDist works mostly in short distance prediction and SDP-Y, SDP-Z, and SDP-S are trained with a target of short distance prediction. For similar reasons, for long distance prediction, we mainly compare SDP-X and SDP-L with PDNET. For MAE values, the smaller the better.

As we see from the Table 2, for long distance prediction $(D_{ij} < 36)$, DeepDist, SDP-Y, SDP-Z, and SDP-S perform much worse than PDNET, SDP-X, and SDP-L. However, SDP-L performs the best among PDNET, SDP-X, and SDP-L in all cases except for CASP13.31 and $S_{ij} > 1$. Between PDNET and SDP-X, the latter performs better than the former. This shows our features and ResNet architecture are better than those of PDNET since both PDNET and SDP-X use the same training and validation proteins and the same sequence library for MSA generation. Our training and validation proteins and MSA generation also make differences since both SDP-L and SDP-X use the same features and ResNet architectures but SDP-L performs better than SDP-X in most cases.

For short distance prediction $(D_{ij} < 16)$ in Table 2, SDP-S performs the best among the 7 prediction methods, regardless of the sequence separation length. Notice that as normally expected, the performance of PDNET, SDP-X, and SDP-L is much worse than that of DeepDist, SDP-Y, SDP-Z, and SDP-S for short distance prediction. Between SDP-Y and SDP-S, the latter performs better than the former. This shows it is better to ignore distances $16\,\AA$ or above when the target is short distance prediction. Notice that SDP-Z is worse than SDP-S but and has a mixed or comparable performance with respect to DeepDist. The performance difference between SDP-S and SDP-Z comes from the training and validation datasets and the MSA generation as both methods use the same features and ResNet architecture. The comparable performance of SDP-Z and DeepDist is interesting. SDP-Z uses about 3500 proteins in its training and validation sets with our input features while DeepDist uses about 6500 proteins in its training and validation sets with many more input features than SDP-Z’s. Moreover, DeepDist generates MSA based on 6 sequence libraries of 2018, while SDP-Z (also all SDP variants and PDNET) does that on 1 sequence library of August 2018. Nevertheles, all these show the effectiveness of our input features and the ResNet architecture over the differences in the protein sequences used in training and validation.

Henceforth, we perform further analysis of SDP-L against PDNET and SDP-S against DeepDist.

**Figure 4:**
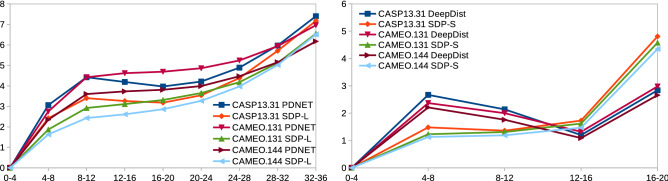
MAE values (y-axis) in various split actual distance ranges (x-axis) for PDNET and SDP-L (left) and for DeepDist and SDP-S (right). The right chart includes the range 16–20 $\AA$ to show the very sharp increasing trend in the later ranges.

Figure 4 shows the MAE values in various actual distance ranges for SDP-L against PDNET and SDP-S against DeepDist in various datasets. As we see, SDP-L and SDP-S obtain smaller MAE values in most cases in all datasets.

**Figure 5:**
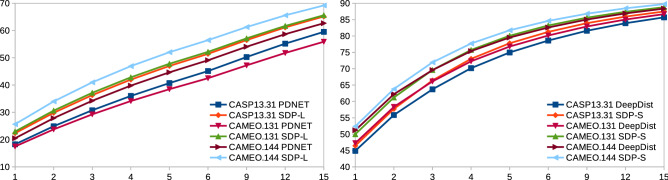
Percentages (y-axis) of residue pairs with actual distances below $36\,\AA$ (left) and below $16\,\AA$ (right) such that those residue pairs have predicted values with absolute errors below various given threshold limits (x-axis).

Figure 5 shows the percentages of residue pairs with short and long actual distances such that those residue pairs have predicted values with absolute errors below various given threshold limits. In this figure, the larger the percentages, the better the performance. As we see from the charts, SDP-L and SDP-S methods perform better than the other methods in most cases.

#### Comparison with LiXu

The LiXu^24^ method is related to another method^60^ but its evaluation is done via contact map prediction accuracy. So we compare mainly with the LiXu^24^ method. As already noted before, LiXu^24^ program is not available to us. So we compare SDP’s performance with the results reported in the article describing LiXu. For this comparison, we use the distance metrics used by LiXu and compute the results for PDNET, DeepDist, and SDP methods. Table 3 shows the comparison of PDNET, DeepDist, LiXu, and SDP methods over CASP13.31 dataset in terms of absolute errors (AE), relative errors (RE), pairwise distance test (PDT) scores, and high-accuracy pairwise distance test (PHA) scores. Note that LiXu^24^ results are reported only for CASP13.31 dataset. Moreover, AE is the absolute difference between the predicted and the native distances while RE is the absolute error normalised by the average of the predicted and the native distances. Furthermore, assuming $R_i$ denotes the fraction of predicted distance with an absolute error less than *i*, PDT is the average of $R_1$, $R_2$, $R_4$ and $R_8$ while PHA is the average of $R_{0.5}$, $R_1$, $R_2$, and $R_4$. Following LiXu^24^, we compute AE, RE, PDT, and PHA for distances less than $15\,\AA$. Nevertheless, as we see from the table, SDP-S outperforms all other methods including LiXu^24^ in all metrics. Moreover, LiXu performs worse than DeepDist, PDNET, and all SDP versions. Moreover, DeepDist is better than PDNET.

**Table 3:** Comparison of PDNET, DeepDist, LiXu, and SDP methods in terms of mean absolute error, relative error PHA and PDT scores over all residue paris in CASP13.31 dataset.

Method	AE	RE	PHA	PDT
PDNET	2.116	0.197	0.494	0.694
SDP-L	1.968	0.184	0.521	0.716
SDP-X	1.999	0.190	0.517	0.713
DeepDist	2.019	0.183	0.514	0.710
SDP-S	**1.672**	**0.154**	**0.553**	**0.750**
SDP-Y	2.104	0.191	0.508	0.701
SDP-Z	1.812	0.167	0.520	0.724
LiXu	4.069	0.241	0.432	0.610

The emboldened values denote the best performances; for AE and RE, the lower the better while for PHA and PDT, the higher the better.

### Comparison of contact maps obtained from distance maps

There is a separate body of research for contact map prediction. Moreover, in this work, our interest is in improving distance map prediction, particularly long range distance prediction, and not contact map prediction at all since distance maps are more informative^11,12^ than contact maps. However, we just want to see what happens if our predicted distance values are converted into contact maps. Predicted distances can be transformed into contact map predictions in the following two ways.

**Via probability method:** Predicted distance $D_{ij}$ can be converted into a contact probability $P_{ij} = \frac{4.0}{D_{ij}}$ if $D_{ij} \ge 4.0$ else 1.0. Then, the top *L* (or *L*/2 or *L*/5) contact probabilities are considered for each protein where *L* is the number of residues in the protein. Next, precision $P_L$ (or $P_{L/2}$ or $P_{L/5}$) is computed for the top *L* (or *L*/2 or *L*/5) contact probabilities assuming two residues are in contact when they are at most $8\,\AA$ apart. This procedure has been used in the literature^12,20,35,44^.
**Direct comparison method:** Predicted distance $D_{ij}$ can be directly compared with the threshold distance $8\,\AA$ and residue pairs having distances $8\,\AA$ or below can be considered to in contact. Then, precision and recall values could be computed.

**Comparison with distance map predictors on contacts.** Using the via probability method described above to compute contacts from distances, Table 4 shows the precision values $P_L$ obtained by various methods when sequence separation lengths are at least 12 or 24. As we see from the table, DeepDist performs the best and SDP-L performs the second best. Using the direct comparision method desribed above to compute contacts from distances, Table 5 shows precision and recall values for all residue pairs. We see that DeepDist has better precision values in 2 out of 3 datasets with SDP-L performing the second best, but SDP-S and SDP-L both have better recall values than the other two methods in all datasets.

**Table 4:** Precision values $P_L$ (%) for top contact pairs when sequence separation lengths $S_{ij} = |i-j|$ are at least 12 or 24.

Test	$P_L$ for $S_{ij} \ge 12$				$P_L$ for $S_{ij} \ge 24$			
Dataset	PDNET	SDP-L	DeepDist	SDP-S	PDNET	SDP-L	DeepDist	SDP-S
CASP13.31	48.77	56.65	**59.60**	54.25	33.93	42.63	**43.76**	39.25
CAMEO.131	48.13	56.92	**58.19**	45.15	37.10	45.58	**47.04**	33.89
CAMEO.144	53.87	61.79	**63.96**	50.33	43.62	51.49	**54.16**	39.20

For $P_L$, the larger the better. The emboldened and underlined values are the best and the second best values respectively.

**Table 5:** Precision and recall values for distance map to contact map direct conversion and for all residue pairs.

Test	Precision				Recall			
Dataset	PDNET	SDP-L	DeepDist	SDP-S	PDNET	SDP-L	DeepDist	SDP-S
CASP13.31	0.873	0.881	**0.905**	0.821	0.738	0.766	0.746	**0.781**
CAMEO.131	**0.865**	**0.865**	0.822	0.750	0.811	**0.835**	0.816	0.834
CAMEO.144	0.890	0.893	**0.917**	0.776	0.810	0.838	0.809	**0.841**

For both metrics, the larger the better. The emboldened and underlined values are the best and the second best values respectively.

In this work, our key focus is to learn long distances between residues having long sequence separation. In LDDT scores in Table 2, SDP-S performs better than SDP-L. However, considering the better MAE of SDP-L over SDP-S for $D_{ij} < 36$ and $S_{ij} \le 12$ and $S_{ij} \le 24$ in Table 2 and better $P_L$, precision, and recall values of SDP-L over SDP-S in Tables 4 and 5, we select SDP-L as our best setting and henceforth only show its performance.

**Comparison with State-Of-The-Art Contact Predictors.** With SDP-L, we compute contact precision values $P_L$, $P_{L/2}$, $P_{L/5}$ for sequence separation lengths at least 12 and 24. In Table 6, we then compare the computed precision values with that of the contact predictors RaptorX-contact^61^, Chen et. al method^62^, and TripletRes^63^. As we see from the table, for $S_{ij} \ge 12$, SDP-L outperforms the other three contact predictors but could not do so for $S_{ij} \ge 24$. Note that all three other methods are specifically designed for contact prediction while SDP-L is primarily designed for distance prediction.

**Table 6:** Precision values for top contacts on CASP13.31 targets.

Method	$S_{ij} \ge 12$			$S_{ij} \ge 24$		
	$P_{L/5}$	$P_{L/2}$	$P_L$	$P_{L/5}$	$P_{L/2}$	$P_L$
RaptorX-contact	0.702	0.527	0.364	0.694	0.567	0.438
Chen et. al	0.665	0.485	0.342	0.707	0.559	0.426
TripletRes	0.770	0.562	0.367	**0.716**	**0.573**	**0.440**
SDP-L	**0.775**	**0.701**	**0.567**	0.678	0.552	0.426

### 3D protein structure construction

**Figure 6:**
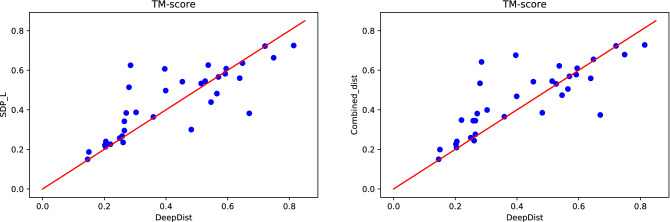
TM-scores of the protein structures obtained by using distace maps predicted by DeepDist and (left) that predicted by SDP-L and (right) that obtained by combining predicted distance maps of DeepDist and SDP-L.

**Figure 7:**
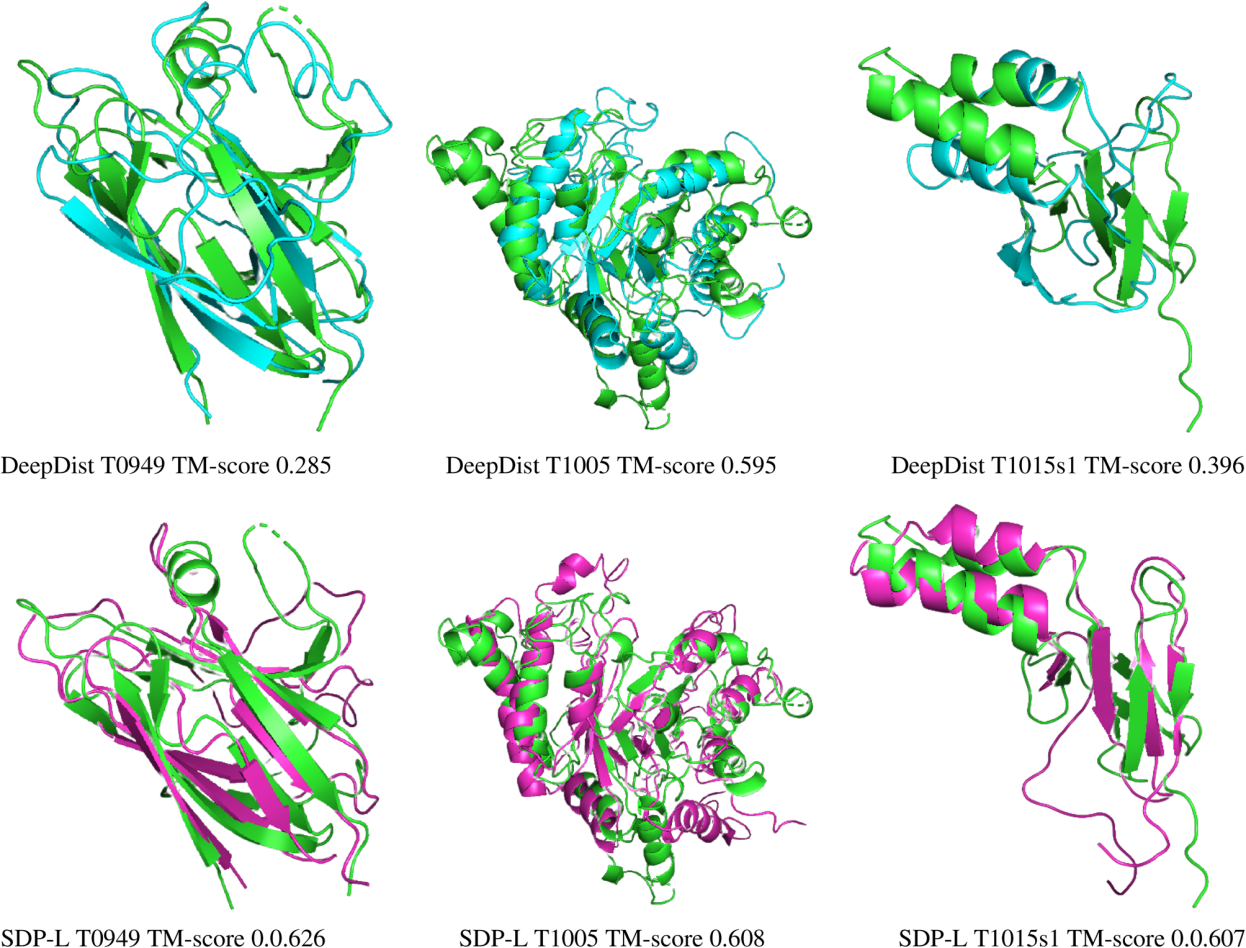
Sample 3D structures of 3 CASP13.31 targets constructed from SDP-L and DeepDist predicted distance maps.

We build three dimensional structures using the distance maps predicted by SDP-L and DeepDist. We cannot do this for LiXu^24^ since its program is not available for us to get its predicted distance maps. For this, we use DFOLD^64^, which has been used by DeepDist^25^ as well. Figure 6 (left) shows the template modeling scores (TM-scores) of the structures obtained for the CASP13.31 proteins. Clearly, SDP-L predicted distances in most cases result in better protein structures than DeepDist predicted distances. Note that DeepDist mainly predicts distances up to $16\,\AA$ while SDP-L predicts up to $36\,\AA$. Further, we create combined distance maps from DeepDist and SDP-L predicted distance maps by taking DeepDist predicted distances when corresponding SDP-L predicted distances are less than 16 otherwise taking SDP-L predicted distances. As we see in Figure 6 (right), this also shows that the combined distance maps result in better structures in most cases than DeepDist predicted distance maps do. Overall, these results show that distances larger than $16\,\AA$ and up to $36\,\AA$ help obtain better three dimensional structures. Figure 7 shows sample protein structures and TM-scores values obtained for three CASP13.31 proteins by using SDP-L and DeepDist predicted distance maps with the same program DFOLD.

## Conclusions

In this paper, for protein inter-residue real distance prediction, we propose deep learning models, which use fewer types of multiple sequence alignment (MSA) and sequence based features than existing such methods. Prediction of inter-residue distances and using such predicted distances in designing protein conformation scoring functions have recently led to considerable progress of protein structure prediction. However, prediction of large distances and distances between residues with long sequence separation length still remains challenging. To overcome these challenges, more and more features have been used in existing distance prediction algorithms. In this paper, we scrutinise the feature space to reduce the types of features being used but at the same time, we strive to improve the prediction accuracy. Using only 2 coevolutionary and 3 non-coevolutionary types of features, we improve mean Local Distance Different Test (LDDT) scores at least by 10% compared to the current state-of-the-art distance prediction methods. Our proposed algorithm is named Scrutinised Distance Predictor (SDP). The SDP program along with its data is available from the website https://gitlab.com/mahnewton/sdp.
